# Intraocular lens power calculation following laser refractive surgery

**DOI:** 10.1186/s40662-015-0017-3

**Published:** 2015-04-02

**Authors:** Christopher Hodge, Colm McAlinden, Michael Lawless, Colin Chan, Gerard Sutton, Aifric Martin

**Affiliations:** Vision Eye Institute, Level 3 270 Avenue Chatwood, Victoria, NSW Australia; Save Sight Institute, University of Sydney, Sydney, NSW Australia; Flinders University, Bedford Park, Adelaide, South Australia Australia; Wenzhou Medical College, Wenzhou, Zhejiang China; School of Optometry and Vision Science, University of New South Wales, Sydney, Australia; University College Dublin, Montrose, Dublin Ireland

**Keywords:** Cataract, Laser refractive surgery, Intraocular lens calculations

## Abstract

Refractive outcomes following cataract surgery in patients that have previously undergone laser refractive surgery have traditionally been underwhelming. This is related to several key issues including the preoperative assessment (keratometry) and intraocular lens power calculations. Peer-reviewed literature is overwhelmed by the influx of methodology to manipulate the corneal or intraocular lens (IOL) powers following refractive surgery. This would suggest that the optimal derivative formula has yet been introduced. This review discusses the problems facing surgeons approaching IOL calculations in these post-refractive laser patients, the existing formulae and programs to address these concerns. Prior published outcomes will be reviewed.

## Introduction

The unfortunate irony for post laser refractive surgery patients has been the difficulty in achieving consistent, accurate refractive outcomes following cataract removal and intraocular lens (IOL) implantation [1,2]. Subsequently, researchers have described various errors in IOL calculation and developed a variety of approaches to overcome the reported refractive surprises [3-5]. Although refractive results have improved significantly over time, it would appear an optimal solution remains elusive, indicated by the significant number of methods available to aid post refractive IOL calculations [6]. The purpose of this review is to identify the issues relating to IOL power calculations and describe the available methods to improve the accuracy of post cataract surgery outcomes. Published results will be discussed to provide an overview of current outcomes.

## Review

There are three main sources of error in IOL calculation for patients who have previously undergone laser refractive surgery: the index of refraction error, the measurement of corneal radius error and the inherent errors within the IOL formulas [7]. These are discussed in more detail below.

### Keratometry-derived errors

Most keratometers measure the central anterior surface of the cornea only. The production of an accurate corneal power value thereby is reliant on several assumptions; firstly that the corneal surface has uniform curvature and power; and secondly that the relationship between the anterior and posterior surfaces remains fixed. The use of the average keratometric index, routinely 1.3375, assumes that these models are adhered to. Ablative laser procedures inherently change the relationship between the anterior and posterior curvature of the cornea, immediately invalidating the above assumptions. Furthermore, the true index of refraction will vary dependent on the amount of laser ablation (and remaining tissue) further impeding the use of a basic or standardized keratometric index [8]. In eyes that have undergone myopic ablative refractive procedures, the resulting keratometry readings obtained by standard keratometry or topography will appear erroneously high, subsequently leading to an underestimation of the final IOL power [1]. Depending on the literature, this value is estimated to represent a difference of between 14% to 25% [9]. The opposite will occur in patients that were previously hyperopic.

This error may be mediated by adjusting the corneal power values through the use of refraction-derived keratometry values or through the use of regression-based formulas [10,11]. Although current literature provides adequate outcomes, the results do remain variable suggesting further validation is still required [12-15]. Bypassing the keratometric index and calculating corneal power by using Gaussian optics formula or ray tracing, based on Snell’s law, may yet provide the optimal approach [12]. Both approaches require the examiner to obtain valid measurements for anterior and posterior corneal curvatures.

A further functional error may occur in obtaining the actual keratometric measurement [7]. Corneal curvature measurements not obtained from the corneal centre may be erroneous [16]. This error has been referred to as the “radius error”. This may reflect both the incapability of some keratometry units (or corneal topographers) to measure the central corneal area and the inability of these devices to incorporate the change to an aspherical cornea produced by the surgery [17,18]. These errors have been ameliorated with the use of diagnostic technology such as ray tracing and with the evolution of lasers using larger effective optical zones. The radius error impacts all post refractive IOL patients, however the risk of significant invalid measurements is increased in patients with small or decentered laser ablations.

It has been suggested that the magnitude of error in IOL power calculations is much lower in previously hyperopic eyes than in post myopic eyes due to the relatively minimal corneal steepening in hyperopic laser in situ keratomileusis (LASIK) and a diminished reduction in the change of refraction index secondary to the corneal steepening [19,20].

### IOL calculation formulas

Most third and fourth generation theoretical formulas share the same basic approach. This includes the primary equation to determine the effective lens position (ELP), based on parameters such as keratometry and axial length. Thereafter, this result is used in the original vergence formula to determine the suggested IOL power [21-23]. Identified by Aramberri, the use of the post laser keratometry values to determine the ELP is flawed [3]. Essentially, anterior chamber depth should not change as a result of the laser surgery. Therefore, using the artificially lower keratometry values will lead to an underestimation of the ELP and resulting IOL power. The proposed “Double-K” procedure, using the preoperative keratometry for the ELP equation and the postoperative keratometry values in the IOL power calculation was derived to overcome this. Subsequently the “Double-K” technique figures in the majority of the calculations proposed [24,25]. This issue was similarly recognized by Holladay and is incorporated into his proprietary software available to surgeons (by checking the “previous RK” box on the IOL Consultant Program) [4]. Of note, within the Holladay IOL Consultant Program, if the pre-laser keratometry values are unknown, a standard value is then used, which has been variably reported as 43.86 and 44.00 in the literature [4,26]. The true value remains under proprietary considerations.

The Haigis-L formula represents an alternative approach to bypass the inherent errors of post refractive subjects. The formula applies a correction curve to the existing measurement to derive the effective corneal power thereby bypassing both keratometer and radius errors [16]. This value is then incorporated into the original Haigis formula for standard eyes. Significantly, the original formula does not use corneal radius as a predictor of IOL position thus further reducing the risk of the formula error.

### Post-refractive surgery derivative formula

To fully describe the historical outcomes of post refractive IOL power formulas, a PubMed database search was performed for relevant literature between 1998 to October 2014. The search included the following keywords; IOL power calculation, refractive surgery, keratometry, LASIK, photorefractive keratectomy (PRK) and radial keratotomy (RK). Two hundred and twenty four articles were retrieved and evaluated. Approximately 70 keratometry or IOL power formulas were found. These were separated into 4 separate sub-headings including history methods, change in manifest refraction, no prior data and others (Table 1). The majority of calculations included in the “Others” column include topography derived keratometry values. Some approaches may have differed minimally from other established formulas and may not have been included.

**Table 1:** **List of known post laser refractive formulas**

**History methods (plus change in manifest refraction)**	**Change in manifest refraction methods**	**No prior data**	**Others**
Camellin*[31]#	Adjusted ACCP [62]°	Actual K (a + p) [59]	CAS-OCT
Clinical History [27]°#	Adjusted Atlas 9000 (4 mm Zone) [34]°	Awwad [61]	Consensus-K [63]
Corneal Bypass [42]°	Adjusted Atlas Ring Values [45]°	BESSt [55]#	Double -K^^^ [3]
Feiz-Mannis [64]°#	Adjusted EffRP [35]°	Canovas Ray Tracing- Aberration [65]	*Corneal topography*
Jarade Index of Refraction*[11]#	Barrett True-K [66]	Ferrara [67]#	Adjusted Flat K
Seitz-Speicher*[31]	Camellin-Calossi [31]	Feiz Myopic [64]	Atlas 0-3
	Chokshi SE [37]	Feiz Hyperopic [64]	Galliei Sim K
	Diehl-Miller [38]	Haigis-L [68]°#	Orbscan Flat Axis
	Diehl-Date-Miller [39]	Hamed [35]	Orbscan Mean and Total Mean Power
	Khalil Regression [40]	Hard Contact Lens [69]#	Orbscan 1.5 mm, 2.00 mm and 2.50 mm Mean Power
	Latkany [41]#	Ianchulev [46]#	Orbscan 4.0 mm Total Optical Power
	Masket [36]°#	Galilei [70]°	Orbscan 5.0 mm Total Axial Power
	Modified Masket [71]°	Geggel Ratio [60]	Pentacam True Net Power (TNP)
	Rosa [72]	Kim [47]	Pentacam Central TNP
	Srivannaboon	Leccisotti [49]	Pentacam 4.5 mm Equiv K
	Stakheev and Balashevich [73]	Mackool [48]#	ORange Intraoperative Wavefront [52]
	Walter [42]#	Maloney [34]#	Ray Tracing (OKULIX Software) [74]
		Maloney-Koch-Wang [34]°	
		Razmjoo Regression [75]	
		Ronje [76]#	
		Saiki (A-P) [77]	
		Saiki (C-P) [56]	
		Seitz-Speicher-Savini [31]	
		Savini-Barboni-Zannini [78]#	
		Shammas [44]°	
		Wang [34]	

^Used in conjunction with other methods to derive IOL power.

*May be used without clinical history information.

°Incorporated into the American Society of Cataract & Refractive Surgeons Post Refractive Calculator.

#Available in the Hoffer-Savini LASIK IOL Power Tool.

Note: references not provided for IOL formulas based on topographic measurement variations.

A discussion of the main formulas and respective refractive outcomes follows.

### Methods based on the knowledge of patient clinical history (refraction and keratometry)

Originally proposed independently by Eiferman and Holladay, the “clinical history method” represented the first method to overcome the intrinsic IOL calculation issues in post laser refractive surgery patients [27,28]. For many years, this method remained the so-called “gold standard” for comparison [29]. The methodology remains relatively simple. The effective keratometry value is calculated by subtracting the change in refraction induced by the treatment from the preoperative mean corneal power. This method effectively bypasses the index of refraction error. The main disadvantage of the clinical history method and similar formulas is the reliance on preoperative keratometry and refractive data. The effectiveness of these methods may be reduced by the possibility of further errors including but not limited to: the use of inaccurate central corneal measurements, variation in measurement units before and after surgery and the potential impact of index myopia [7,9,30]. Practically, the follow-up required to obtain the data may be particularly cumbersome if the patient had their refractive surgery elsewhere or a number of years previously. In a large study, Wang et al. found that formulas requiring both the preoperative refraction and keratometry values performed poorly compared to other available methods. Results showed significant variability with high IOL prediction errors and relatively low percentages of outcomes within ± 0.5 D and ± 1.0 D [29]. This has been mirrored in subsequent studies and these methods should no longer be used [31-33].

### Methods based on the knowledge of change in manifest refraction

Several approaches aim to bypass the need for preoperative keratometry and represent valid alternatives.

A number of methods propose applying a correction, based on the change in refraction, to the postoperative keratometry value. This value is then appropriately inserted into the Double-K formula to provide the final IOL power [34,35]. The “Adjusted Effective Refractive Power (EffRP)” and “Adjusted Atlas 9000 (4 mm Zone)” methods are commonly used examples available through the American Society of Cataract and Refractive Surgeons (ASCRS) IOL Calculator. These methods rely on the availability of the particular topographical unit and require the examiner to directly assess the measurement to confirm the quality of the reading.

Masket and Masket recognized the difficulty in obtaining accurate postoperative corneal measurements and effectively bypassed these potential errors by creating a formula based on the change in laser correction [36]. The authors determined that the chief corrective factor in post-refractive patients was the amount of pre-ablative myopia. Subsequently, a value based on a simple regression formula deriving the change in manifest refraction, was added to the standard IOL calculation. Other authors have undertaken similar approaches with basic variations of the regression formulas [37-41]. Walter et al. described an even simpler approach: by assuming the patient never had myopic laser surgery and replacing the standard IOL target with the pre-laser amount of myopia [42]. Although the authors reported excellent results, the initial study has been criticized by the use of a small sample size to derive the formula.

The risk of index myopia related errors remains considerable for methods based on the change in manifest refraction.

### Methods based on no prior data

Common to all previous formulas is the requirement for preoperative keratometry and/or refraction data. This is often unavailable to surgeons and alternative approaches therefore remain necessary. Formulas based on postoperative information serve either to re-measure or recalculate the current keratometry values prior to entry into existing IOL calculations.

The over-refraction of a hard contact lens has been described previously [30,43]. This effectively re-measures the corneal curvature rather than providing a recalculated value. This approach has been limited by technical and time constraints. Difficulty in achieving an adequate refraction in patients with poor visual acuity further reduces the effectiveness of this method.

Shammas et al. previously described a simple equation modifying post-laser keratometry values to determine the corrected corneal power to be used in IOL calculation formulas [44]. Other researchers have taken similar approaches. The Maloney and Koch-Maloney methods convert the post-laser keratometry values from corneal topography to the exact power present at the anterior corneal surface and then add an average negative power value for the posterior corneal surface [45]. These latter formulas have been based on values obtained by the Atlas topography (Zeiss, Germany) and thereby remain of limited value to practices without this unit.

As described earlier, the Haigis-L formula bypasses the various errors through the use of a correction formula then applied to the standard Haigis formula. The relative availability of the Haigis-L formula across several platforms including both instrument and web-based programs, has led to the formula rapidly becoming a focus of many comparative studies albeit with varying results [46,47].

Mackool et al. suggested an alternative approach [48]. The cataract is first removed and the patient is required to wait for an hour before an aphakic refraction is undertaken. An algorithm is then applied to the refraction to determine the true IOL power to be inserted. Ianchulev and Leccisotti also described “on the table” approaches with reasonable postoperative outcomes [49,50]. Sheppard, however, compared all three intraoperative aphakic formulae with mixed results [51]. The author suggested that intrinsic differences within the formulae determine the most appropriate use in patients, that is, both the Leccisotti and Ianchulev formulae appear to provide better results for posterior IOL positions and the Mackool algorithm more appropriate for anterior chamber IOLs. Intraoperative reforming of the anterior chamber either with balanced salt solution or visco-surgical devices and variable refractive indexes remain significant obstacles for these methods achieving consistent postoperative outcomes [49-51]. More recently, the use of intraoperative wavefront aberrometry has been described to further refine outcomes [52]. Results using the WaveTec Intraoperative Wavefront aberrometer were compared with several established formulae in post refractive patients. The WaveTec readings were more accurate than predictive formulae, most often predicting to within ± 0.5D of emmetropia. Tellingly however in this study, no method was able to achieve this accuracy more than 50% of the time, which highlights the relative inaccuracy of the post refractive formulae. Ianchulev and co-authors recently described more optimistic results with intraoperative aberrometry. They detailed results achieved with the Optiwave Refractive Analysis (ORA) System wavefront aberrometer that appeared to surpass those with comparative preoperative methods of IOL calculation [46].

### Ray tracing

The principles of ray tracing suggest that using this method for IOL calculations may provide more accurate, reproducible results compared to existing alternate keratometric methods. Ray tracing technology is currently available in many topographical systems although results may be enhanced with the addition of external computational programs such as Okulix (Oculix, Dortmund, Germany). Savini et al. indicate that ray-tracing avoids the systemic issues involved with post refractive cases [53]. Calculating the refracted ray at both anterior and posterior surface avoids the use of the average, fictitious corneal refractive index. The ability of the ray tracing software to be performed over any corneal diameter minimizes potential instrument and thereby radius error. Finally, formula errors are avoided as the IOL position may be calculated without respect to the single anterior curvature value [53]. Hoffmann et al. suggested that although the prediction accuracy of ray tracing remains only comparable to third-generation formulae, the accuracy in abnormal eyes, those with long or short axial length and those with prior refractive surgery is improved [54]. The results obtained by Savinni and co-authors in recent papers further highlights the potential of using values based on ray tracing principles [12,17,53].

### Available sources of calculations

Although a number of formulae may now provide improved refractive outcomes in post laser refractive surgery patients, it remains a difficult and time-consuming action to derive the IOL powers using multiple formulae. This task however, has been made significantly easier with the advent of several available web- or app-based programs.

Developed with the support of the ASCRS, the ASCRS Post Refractive Calculator (www.iolcalc.org) remains an efficient method for obtaining multiple IOL formulae. The program utilizes several referenced formulas and provides an average of all available methods. Recognizing the increasing reliance on non-historical methods, the website recently introduced a further value representing the average of all non-historical methods. The formulae from the ASCRS website have been used in several studies and appear to provide consistent, reliable outcomes [24,25,29,52].

The Hoffer-Savini LASIK IOL Power Tool represents a similar approach, albeit through the use of a downloadable spreadsheet (available from www.iolpowerclub.org/post-surgical-iol-calc). The user enters the available keratometry and biometric values from which the spreadsheet provides the recalculated corneal power values to use in subsequent IOL formulae. The spreadsheet also calculates the IOL power directly through several available formulae. Although the IOL Power Tool replicates some of the formulae in use with the ASCRS web tool, the use of alternative formulae may provide further information for the surgeon.

Several other websites or applications remain available albeit without the range of the aforementioned sites. The Asia Pacific Association of Cataract and Refractive Surgeons (www.apacrs.org) provides the Barrett True-K formula for post-refractive IOL patients. The McCarthy Post Refractive IOL Calculator (www.mccarthyeye.com/post-refractive-iol-calculator) provides several relevant formulae based on the outcomes of the authors’ prior study of 173 post-LASIK eyes [32]. Several applications are available for use with existing iOS or Android devices. These include the Eye Pro application, which utilizes the BESSt post refractive IOL formula and the PAK post refractive IOL formula. [**Note:** the BESSt formula requires the input of keratometry values obtained by the Pentacam topography unit (Oculus Pentacam, Germany) [55-57]].

## Results

Within the UK National Health Service, figures of within ± 0.50 D for 55% and within ± 1.00 D for 85% have been used to provide a standard for refractive outcomes following cataract surgery in normal eyes [58]. While this gauge underestimates the visual outcomes now expected, it supplies a benchmark standard that has been used in previous studies [29,32,39]. Table 2 delineates comparative results from past papers evaluating a variety of formulas in post laser vision correction cataract procedures.

**Table 2:** **Published post refractive IOL calculation outcomes within ± 0.5 D and ± 1.0 D of target**

**First author (Year)**	**Method**	**Percentage within ± 0.50 D**	**Percentage within ± 1.00 D**	**Number of patients assessed overall**
Savini (2013) [12]	Overall	60.7%	85.7%	28
DeMill et al. (2011) [25]	Ocular MD Calculator Average	76%	90%	21
Hamed (2002) [35]	EffRP Adjusted	70%	94%	100
Date et al.(2013) [39]	Diehl-Miller-Date Formula	49%	93%	23
Masket (2006) [36]	Masket	93.3%	100%	30
Hu (2010) [59]	Actual K (a + p)	80%	100%	10
Ianchulev (2014) [46]	Intraoperative Refractive Biometry	67%	94%	246
Geggel (2013) [60]	Geggel Ratio /Haigis	78%	100%	34
	Consensus	70%	93%	
Saiki (2013) [77]	Anterior-Posterior Method	46.4%	75%	28
Saiki (2013) [56]	Central-Peripheral Method	48%	68%	25
Saiki (2014) [74]	Ray Tracing	41.7%	75%	24
Savini (2014) [53]	Ray Tracing	71.4%	85.7%	21
Canto et al. (2013) [52]	ORange	39%	60%	53
Yang (2013) [24]	Best Performing	58%	90%	62
Wang et al.(2010) [29]	Best Performing	67%	90%	72
McCarthy et al.(2011) [32]	Best Performing	58.8%	84.3%	173
Tang (2010) [33]	OCT Guided	78%	--	27
Arce (2009) [79]	Orbscan Central 2 mm TMP	53%	78%	77
Qazi (2007) [80]	Orbscan 4 mm TOP	80.9%	95.2%	21 (back calculated)
Shammas (2007) [81]	Shammas	--	93.3%	15
Javadi (2012) [82]	Adjusted Flat K 3 mm	44.4%	61.1%	18
Cai (2011) [83]	Orbscan Mean Power	48.4%	80.6%	62

The range for values occurring within ± 0.50 D and ± 1.00 D remains highly variable. The lowest percentage is reported by Canto et al. [52] at 39% and 60% respectively, for their small series detailing the use of an intraoperative wavefront aberrometer. This would suggest that recording the simple intraoperative value does not take into account the impact of all surgical variables. Conversely, Masket and Masket reported 93.3% and 100% of patients within ± 0.50 D and ± 1.00 D respectively of the intended correction [36]. Both Geggel and Hu similarly reported 100% of their patients within ± 1.00 D following surgeries in their respective cohorts [59,60]. Although Masket and Masket’s original paper was based on a relatively small sample size, which may have biased the final outcome, importantly the success of the formula has replicated with consistently good outcomes in further studies [31,32,61].

A criticism of most post-refractive IOL formulae remains the relatively low numbers used to derive the accompanying outcomes. The range amongst the listed publications remains significant from a minimum of 10 patients to a maximum of 246 (median of 27 patients) [46,59]. The availability of formulae and relative number of post refractive patients proceeding to surgery would suggest that further audits might provide additional important information to assist surgeons in choosing the most appropriate formulae. Similarly, it might provide a platform for a revised methodology.

## Conclusion

The optimal correction of post-refractive IOL patients represents an ongoing concern for surgeons. The use of available programs and existing formulas serves to improve upon prior results however literature suggests that consistent outcomes similar to those obtained in virgin eyes currently remains out of reach. The continued audit of post-refractive IOL outcomes, the development of corneal imaging technology, and improved intraoperative wavefront aberrometry may provide the best opportunity for surgeons in the short to medium term.
